# The Cost of the Hospital Nurse

**Published:** 1909-06-26

**Authors:** 


### Nursing Administration.

### THE COST OF THE HOSPITAL NURSE.

The cost of the nursing department is intimately bound up with its efficiency. Strange to say, lavish expenditure is rarely, if ever, found marching hand in hand with a high general level of performance, and although the best results can never be attained where a department is starved of the things necessary for its proper expansion, yet the all-pervading supervision which ensures that everyone in the establishment is doing the best work of which they are capable, exhibits itself most surely in keeping down unnecessary expenditure. Extravagance is the unfailing indication of feeble administration, which will not long be confined to questions of the purse.

The recent edition of *Burdett's Hospitals and Charities** has an exceptionally full chapter on the cost of the nursing department in 1907, the year to which all the figures in the volume relate. The figures collected through the courtesy of the matrons and secretaries throughout the country enable a good idea to be formed in regard to the cost of both nurses and nursing. Comparison is rendered possible by adding the amounts spent on nurses' board, uniforms, laundry, salaries, and pensions or premiums. This total is divided by the average number of nurses in residence, to give the average cost of each nurse to the institution, and is divided by the average number of occupied beds to give the average cost of nursing per bed. It must be at once explained that this estimate of cost necessarily fails to take into consideration a number of additional expenses incurred on behalf of the nursing staff in the nursing home. These collective sums include the cost of service, rent, taxes, water, coal, lighting, domestic upkeep, furnishing, repairs, etc., etc. They are omitted in the calculation, because hardly any hospitals keep the accounts of the nurses' home separate in these particulars from those of the hospital. It is much to be regretted that this is so, for the nurses' home is now almost invariably, in large hospitals, an entirely separate establishment, and its budget ought to be made out in distinct form so that managers could clearly ascertain how much it costs, and how much it ought to cost. At one or two exceptionally well administered hospitals the accounts of the nurses' home are available in separate form, and the "Annual" quotes that of Guy's Hospital, from which it appears that an average sum of £7.3 per head was expended on these collective items in 1907, and the sum of £6.9 in 1908. As these amounts do not include any expenditure on rent, the Nurses' Home at Guy's Hospital being built on the hospital premises, it may be safely reckoned that an average amount of at least £10 should be reckoned on to the total cost of nurses per head, in the following estimates. The figures given furnish, nevertheless, however incomplete, a good test of economy. If some items in the household are kept at a low level it is not likely that others will be excessive. If the cost of the housekeeping for board is reasonable, it is improbable that coal and gas are wasted, or that too many servants are employed. To the practised eye of the critic it is sufficient to glance at one page of a book to gauge its general style, and it is true of the housekeeper that she may be tested by examination of any one of her departments.

For purposes of comparison it is more satisfactory to take the average cost of each nurse, rather than the average cost of nursing per bed. It should be quite possible, by taking pains, for all hospitals in their own class to arrive at very much the same average in respect of the cost of their nurses. Salaries are much on the same level in hospitals presenting the same description of work. Board is purely a question of management. Uniform and laundry differ but little. But the wide distinctions in administration between one hospital and another render it impossible to fix an exact average of the number of nurses required in proportion to the beds, and hence, when the millennium of hospital management shall arrive, and perfect economy reign in every department, the average cost of nursing per bed will still show wide distinctions. The following table shows the average cost of the nurses per head, arranged in ascending order, for the leading hospitals with medical schools in the United Kingdom. An addition of from £1 to 30s. must be made for hospitals with an asterisk, as their figures do not include the cost of uniform:

| | Daily Average Number of Occupied Beds | Total Nursing Staff | Average Cost of Nurses per Head £ |
|---|---|---|---|
| London :— | | | |
| Guy's ... ... ... | 515 | 251 | 35.6 |
| University College ... | 244 | 103 | 41.6* |
| Royal Free ... ... | 138 | 62 | 44.1 |
| King's College ... ... | 189 | 87 | 45.6 |
| St. Mary's ... ... | 257 | 112 | 48.1 |
| St. Bartholomew's ... | 575 | 288 | 50.7 |
| Middlesex ... ... | 270 | 117 | 52.3 |
| St. Thomas's ... ... | 480 | 192 | 53.0 |
| St. George's ... ... | 319 | 147 | 53.3 |
| London ... ... ... | 792 | 441 | 52.7* |
| Provincial :— | | | |
| Oxford Radcliffe Inf.... | 116 | 41 | 35.7 |
| Birmingham General | 305 | 106 | 39.5 |
| Birmingham Queen's | 121 | 44 | 40.3† |
| Cambridge Addenbrooke's ... ... | 107 | 37 | 42.1† |
| Leeds General Inf. ... | 368 | 95 | 42.9† |
| Scottish :— | | | |
| Dundee Royal Inf. ... | 276 | 94 | 35.5 |
| Edinburgh Royal Inf. | 832 | 272 | 37.6 |
| Glasgow Western Inf. | 544 | 176 | 39.3 |
| Aberdeen Royal Inf. | 226 | 81 | 38.3*† |
| Glasgow Royal Inf. ... | 593 | 170 | 45.3 |
| Irish :— | | | |
| Belfast Royal Victoria | 223 | 77 | 39.8 |

* Minus cost of uniforms. † Board estimated.

It will be noted that there is a difference of £17.1 between the maximum and minimum in London, of £7.2 in the provinces, and of £9.8 in Scotland. It is certain that were all the collective items of the nursing homes available for comparison these differences would be much wider. As they stand they cannot be considered satisfactory.

* *Burdett's Hospitals and Charities*, 1909, being the Yearbook of Philanthropy and the Hospital Annual. (The Scientific Press. Price 7s. 6d. net.)

